# Preclinical evaluation of passive disinfection caps with a long-term catheter for the prevention of catheter-related bloodstream infection in pediatric cancer patients

**DOI:** 10.3205/dgkh000391

**Published:** 2021-05-31

**Authors:** Arne Simon, Wolfgang Fahrendorf, Guido Hitschmann

**Affiliations:** 1 Pediatric Oncology and Hematology, Children’s Hospital Medical Center, Saarland University Hospital, Homburg/Saar, Germany; 2 3M Medical Solutions Division, Health Care Business Group, 3M Central Europe Region, 3M Deutschland GmbH, Neuss, Germany; 3 Medical Solutions Division Laboratory, Europe, Middle East and Africa, 3M Deutschland GmbH, Neuss, Germany

**Keywords:** bloodstream infection, central venous catheter, pediatric cancer patients, scrub the hub, passive disinfection device

## Abstract

The use of passive disinfection devices (disinfection caps) may be a beneficial part of a maintenance care bundle, aiming at the prevention of catheter-related bloodstream infections in pediatric cancer patients. This preclinical *in vitro* investigation tested the visual and mechanical integrity of a Broviac™ catheter hub after simulation testing with 122 3M™ CurosTM Stopper Disinfection Caps for Open Female Luers repeatedly attached and removed over 6 months. We found that these catheter hubs were compatible, fully operational, and airtight with use of 3M Curos stopper caps after 6 months of use with 122 caps per catheter hub.

## Zusammenfassung

Der Einsatz von Verschlusskappen, die Isopropanol zur lokalen Desinfektion des Katheterhubs freisetzen, kann bei Kindern mit Krebserkrankung ein wichtiger Bestandteil des Präventionsbündels von Infektionen werden, die vom Gefäßkatheter ausgehen. In dieser präklinischen *in vitro* Untersuchung wurde über einen Zeitraum von 6 Monaten (122-facher Wechsel der Verschlusskappe) überprüft, ob 3M™ CurosTM Desinfektionskappe für offene weibliche Luer-Lock Verbindungen die optische oder mechanische Integrität der Hubs beeinträchtigt. Die Ergebnisse zeigen, dass diese Hubs durch die langfristige Anwendung des Medizinprodukts nicht beeinträchtigt werden.

## Introduction

Long-term central venous access devices (CVAD), such as a Broviac Catheter [1] or a Hickman Catheter [2] are indispensable tools in pediatric oncology centers (POC), facilitating the administration of cytotoxic chemotherapy, parenteral nutrition, and supportive medication, such as antibiotics, antifungals, analgesics, and blood products. In addition, these long-term catheters allow the frequent, painless sampling of blood for laboratory or microbiological investigations. The use of CVADs has been linked to catheter-associated bloodstream infections (CABSI), which may derive from the catheter entry site (extraluminal source) or the hub or inner surface of the catheter (intraluminal source) [3]. Most POCs have implemented maintenance-care bundles to prevent these adverse events [4], [5].

Frequent manipulation of the catheter hub or any other access point (such as 3-way stopcocks) increases the risk of catheter colonization and subsequent CABSI. In this regard, national [6] and international guidelines [7], [8] concerning maintenance care of vascular catheters recommend hand disinfection before any manipulation and emphasize the necessity of disinfecting the catheter hub (or any other access point) [9](9) before each manipulation. (Some POCs use needleless connection devices, but a significant impact of these devices on the prevention of CRBSI has not been confirmed and the membrane of the device still needs to be disinfected.) 

Hub disinfection of a central venous access catheter can be accomplished by three different methods:

1. Scrubbing the hub with a sterile cloth, which contains antiseptics (such as isopropanol with or without chlorhexidine or octenidine; scrub the hub) [10], [11], [12].
2. Application of the antiseptic as a spray, while holding the hub on a sterile gauze pad [13].
3. Using sterile caps which release IPA after they have been screwed on the Luer lock hub (passive disinfection devices).

To be effective, methods 1 and 2 need a defined dwell time for the antiseptic (e.g., at least 15 seconds). One caveat of infection prevention in clinical practice is that each procedure depends not only on knowledge (education) and skills (training), but also on adherence to recommendations/guidelines of the responsible healthcare worker [14]. Taking limited personnel and time into consideration, any time-consuming procedure may not be consistently followed in a busy unit [15]. 

External scrubbing of a female Luer lock with a cloth is not an effective method to disinfect the inner surface of the connection [16], [17]. In pediatric oncology, this is of particular interest, since health care workers often use central venous access catheters for blood sampling, and any blood residue must be removed thoroughly.

Method 2 is recommended by the German Commission for Hospital Hygiene and Infection Control (KRINKO) as one feasible alternative [18], but it may cause antiseptic inhalation exposure to the patient and the health care worker. 

From a clinical perspective, the use of passive disinfection devices (method 3) intuitively appears to be an attractive alternative. Recent studies including cancer patients demonstrated the feasibility and effectiveness of this approach [15], [17], [19], [20], [21], [22], [23], [24], [25]. Referring to a recent meta-analysis [26], the available studies still are heterogeneous and all have certain limitations (e.g., interrupted time-series investigations instead of prospective randomization). The KRINKO advises attending physicians to consider the use of passive disinfection devices in high-risk clinical units with frequent catheter manipulation and high CABSI rates [18].

Before the implementation of method 3 in the Pediatric Hematology and Oncology unit of the Children’s Hospital Medical Center at Saarland University Hospital, a literature search and personal communication with the manufacturer of the Broviac™ catheters revealed that no preclinical data were available to confirm that the hub of these catheters tolerates the long-term use of a passive, IPA-containing disinfection device in terms of stability and mechanical integrity. In an individual patient, a central venous access catheter is often used for 6 to 12 months until intensive anticancer treatment is completed. Thus, the central venous access catheter must tolerate the use of the passive, IPA-containing disinfection device in the longer term. The preclinical investigation presented here was performed to confirm this before passive disinfection devices were integrated as a routine component of our preventive bundle [4].

## Methods

In our unit, Broviac™ catheters are flushed (with ready-to-use syringes containing 10 ml of sterile 0.9% sodium chloride) and locked with heparin (2–3 ml; 100 IE/ml, 5 ml *single use only* vials) at least once a week. During their intensive treatment period, many patients must visit the pediatric cancer outpatient clinic twice a week for clinical examination and laboratory investigation.

In this regard, the *in vitro* study contained two changes of the passive disinfection cap (3M™ Curos™ Stopper Disinfecting Cap for Open Female Luers) per week. To simulate long-term use, the *in vitro* investigation was continued for 6 months. The materials used were three 6.6 F Broviac™ CV Catheter repair kits 1.0 mm Lumen (BARD Access Systems, Inc., Salt Lake City, UT, USA; Lot REDS2687, expiration date: 2022-04) and 3M™ Curos™ Stopper Disinfecting Cap (Teal) for Open Female Luers (3M Health Care, St. Paul, MN, USA; Lot 6523027, expiration date: 2021-06-03, 6542554, expiration date: 2021-09-26). The white hubs of three catheter repair kits were tested throughout this experiment. Curos stopper caps were placed on the hubs, following the instructions for use. Initially, the caps were changed five times per day, leaving at least 90 minutes between the changes. This sequence of events was performed Monday through Friday for two consecutive weeks. For an additional 24 consecutive weeks, the caps were changed three times per week (Mon, Wed, Fri). The caps were left on the catheter hubs between the changes. The test samples were stored at room temperature (20°–25° C). After the test period of 26 weeks, the catheter hubs to which Curos stopper caps were attached were visually inspected using a microscope to identify any type of mechanical degradation such as crazing, hairline cracks, chips, or warping. The catheter hubs were then exposed to 300 mbar air pressure and soaked in a water bath to verify air tightness. Eventually, each catheter hub tested had a total of 122 caps attached and removed during the 6-month test period.

## Results

No visible differences were observed between catheter hubs treated with 3M Curos stopper disinfection caps. The catheter hub-cap connections were fully operational and airtight after 6 months of testing. In summary, from the results of this testing, it can be concluded that the catheter hubs were compatible with use of 3M Curos stopper caps through 6 months of use with 122 caps per catheter hub. The official test protocol is available as an online supplement to this article.

## Discussion

This *in vitro* study confirmed that the mechanical integrity of the tested Broviac™ catheter model’s catheter hub was not compromised by regular use of 3M Curos stopper caps over a six-month period. 

Formally, it should be acknowledged that the results reported here apply only to the mentioned catheter hubs with the specific lot number. On the other hand, there is no reason to assume any differences between the standard materials used in Broviac™ catheters of the same manufacturer with different lot numbers.

For clinical practice, this result is very important. These catheters are implanted by a pediatric surgeon in an operation theater and remain in place for 6 to 12 months or even longer. Theoretically, the availability of Broviac™ repair sets allows replacement of the most distal part of the catheter (including the hub). Unfortunately, this repair requires extensive manipulation, results in a diminished stability of the catheter against traction force, and the defects liability of the medical device extends from the manufacturer to the physician who performs the repair. Therefore, maintaining the integrity of the components of the Broviac™ catheter and avoiding repairs is advantageous.

The German Commission for Hospital Hygiene and Infection Control states that – in this clinical field – only materials that tolerate alcohol disinfection should be used (e.g., 3-way stopcocks) [18]. Other manufacturers may use the method presented here to investigate their medical devices accordingly. Finally, this investigation enabled us to include the IPA-containing passive disinfection cap into our routine maintenance bundle for the prevention of CABSI (4).

## Notes

### Competing interests

GH and WF are employees of 3M Deutschland GmbH, Neuss. 3M develops and sells medical devices for the prevention of catheter-related infections, including the passive disinfection device investigated in this study.

AS has received honoraria as a speaker and trainer in educational sessions concerning the prevention of catheter-related bloodstream infections (2019, 2020) and for the participation in an expert advisory board on this topic (2020) from 3M. 

Since 2004, AS has been an appointed member of the Commission for Hospital Hygiene and Infection Control, affiliated with the Robert Koch Institute Berlin and coordinates the German recommendations for the prevention of vascular catheter-related infections. AS is a member of the management board (2^nd^ chairman) of the German Society for Pediatric Infectious Diseases.
